# Classification of Alpine Skiing Styles Using GNSS and Inertial Measurement Units

**DOI:** 10.3390/s20154232

**Published:** 2020-07-29

**Authors:** Christina Neuwirth, Cory Snyder, Wolfgang Kremser, Richard Brunauer, Helmut Holzer, Thomas Stöggl

**Affiliations:** 1 Salzburg Research Forschungsgesellschaft m.b.H., Techno-Z III, Jakob-Haringer-Straße 5, 5020 Salzburg, Austria; wolfgang.kremser@salzburgresearch.at (W.K.); richard.brunauer@gmx.at (R.B.); 2 Department of Sport and Exercise Science, University of Salzburg, Schlossallee 49, 5400 Hallein/Rif, Austria; cory.snyder@sbg.ac.at (C.S.); thomas.stoeggl@sbg.ac.at (T.S.); 3 Athlete Performance Center—Red Bull Sports, 5020 Salzburg, Austria; 4 Atomic Austria GmbH, Atomic Strasse 1, 5541 Altenmarkt, Austria; helmut.holzer@atomic.com

**Keywords:** accelerometer, decision trees, gradient boosted trees, gyroscope, random forests, sensors, ski, sports analytics

## Abstract

In alpine skiing, four commonly used turning styles are snowplow, snowplow-steering, drifting and carving. They differ significantly in speed, directional control and difficulty to execute. While they are visually distinguishable, data-driven classification is underexplored. The aim of this work is to classify alpine skiing styles based on a global navigation satellite system (GNSS) and inertial measurement units (IMU). Data of 2000 turns of 20 advanced or expert skiers were collected with two IMU sensors on the upper cuff of each ski boot and a mobile phone with GNSS. After feature extraction and feature selection, turn style classification was applied separately for parallel (drifted or carved) and non-parallel (snowplow or snowplow-steering) turns. The most important features for style classification were identified via recursive feature elimination. Three different classification methods were then tested and compared: Decision trees, random forests and gradient boosted decision trees. Classification accuracies were lowest for the decision tree and similar for the random forests and gradient boosted classification trees, which both achieved accuracies of more than 93% in the parallel classification task and 88% in the non-parallel case. While the accuracy might be improved by considering slope and weather conditions, these first results suggest that IMU data can classify alpine skiing styles reasonably well.

## 1. Introduction

The increasing miniaturization and efficiency of sensing hardware enable novel applications for low-friction activity monitoring. Today, a plethora of wearable consumer devices is available (see [1]). One category of sensors, the inertial measurement unit (IMU), is used in many consumer electronics to track motion and orientation in three-dimensional (3D) space [2]. They are a popular choice for health and sports applications, where they are mainly used to track equipment (e.g., [3,4]) and the human body (e.g., [5,6]).

This work classifies alpine skiing turns into four alpine skiing styles: snowplow, snowplow-steering, drifted and carved based on a global navigation satellite system (GNSS) and IMU data. These four skiing styles can be better understood as falling into one of two broad categories: Parallel and non-parallel. In parallel skiing, both skis act parallel to one another, while during non-parallel skiing, the skis are placed in a wedge [7].

This wedge position, commonly called snowplow, prevents acceleration while the skis are in the fall-line and is therefore a technique used frequently by skiing instructors for beginner skiers. As skiers develop more skills, they may initiate the turn using a snowplow but then complete the turn with the skis parallel. This technique is referred to as snowplow-steering and is the bridge between parallel and non-parallel turns [7]. In the drifting technique, the skier actively steers the skis throughout the turn. This can be accomplished by creating both an angle between the ski and snow (roll or edge-angle) and an angle between the ski and ski trajectory (yaw or steering angle) [8]. Finally, carving refers to the parallel technique in which the ski’s tip creates a groove in the snow which is followed by the rest of the ski, resulting in a self-steering effect [9]. Carving is the more dynamic of the two parallel styles and, generally, this technique used by competitive skiers during racing [10].

These four skiing styles are easily distinguishable by simply looking at the athlete’s posture, the skis’ orientations and their effect on the snow. However, an IMU-based system does not record these visual phenomena—at least, not directly. They record 3D acceleration and angular velocity, and any further features have to be derived in a feature extraction step (c.f. [11], step 3). Such derived features with relevance to alpine skiing include estimated inclination angle [12] and ski symmetry.

IMUs have already been used widely in the field of skiing to estimate skier pose or kinetics [13]. Estimated parameters range from whole body pose [14], trunk orientation [15] or center of mass kinematics [16] to quantification of vibration load on the lower back [17]. While these works present useful tools for performance evaluation or injury risk assessment, they are not feasible for implementation in a wearable system prepared for “plug-and-play” use. Current IMU based methods require calibration, large sensor-networks and offline post-processing in order to produce motion data. Recent work by this group has developed a framework and method for the automated online assessment of alpine skiing turns using a wearable system of IMUs [3,11,18].

Previous works have already used machine learning for classification problems in the context of winter sports. Various authors tried to classify IMU data into cross-country skiing techniques. Rindal et al. [19] used neural networks, while Jang et al. [20] compared convolutional neural networks and long-short term memory based deep learning classification models with k-nearest neighbor classification. Stöggl et al. [21] used Markov chains of Gaussian distributions. Groh et al. [22] classified freestyle snowboarding tricks using inertial-magnetic measurement unit data and naïve Bayes classifiers. There has been work done to use machine learning techniques to classify alpine skiing activities using IMUs [23,24]. However, the algorithm proposed by Han et al. [23] only classified between different activities, such as riding a chairlift, skiing groomed snow or skiing in slush; it did not classify within a skiing activity itself. Pawlyta et al. [24] focused on the analysis of skiing activity recognition, such as the recognition of the turn, the leg lift, the ski orientation and body position.

The purpose of this work was to develop an IMU based classifier, feasible for online implementation in a wearable system, to differentiate between skiing styles. We first conduct a pre-classification into parallel and non-parallel, perform a feature extraction, compare models with a variety of performance metrics and finally identify the most important features distinguishing between the four skiing techniques. The classified skiing turns can be further implemented within a framework such as the extended activity recognition chain (eARC) proposed by Brunauer and colleagues [11].

## 2. Materials and Methods

### 2.1. Sensors

The wearable system used to produce the data consisted of two IMUs (configuration: 2.5 × 3 × 1 Hz 0.83 mm, ±8 g and ±500 dps full scale resolution, board by Movesense [25]) mounted to the upper posterior cuff of each ski boot using a custom housing and strap. This housing was directed, i.e., it was impossible to attach the sensor to the boot in any other than the intended way. It also applied clamping pressure to the sensor, which together with the strap ensured that the sensor was not dislocated during skiing.

**Figure 1:**
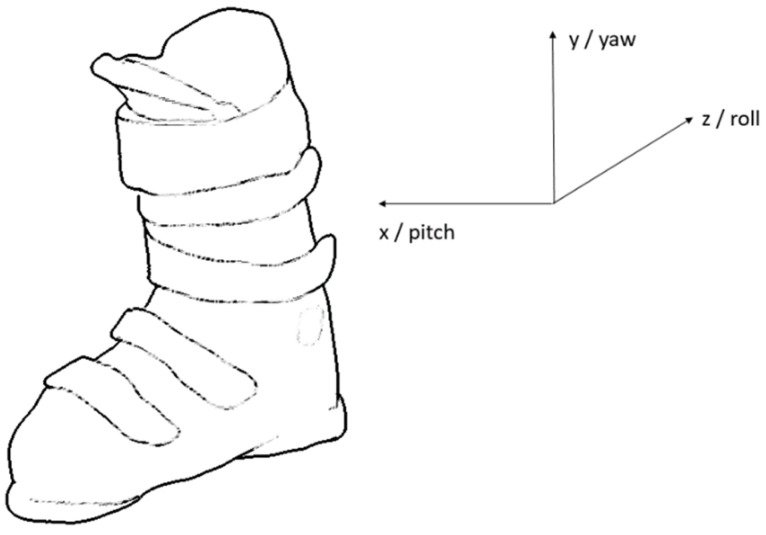
Inertial measurement unit (IMU) axes (accelerometer and gyroscope).

The Y axis of the IMU was aligned with the vertical axis of the boot pointing superiorly, the X with the lateral axis pointing to the right and the Z with the roll axis pointing posteriorly (see Figure 1). The output data rates of both 3D accelerometer and gyroscope were set to 833 Hz. The accelerometer signal was filtered by an analog anti-aliasing low-pass filter and again after analog/digital conversion by a digital low pass filter (cutoff 416.5 Hz). The gyroscope signal was filtered by a digital low-pass filter (cutoff 245 Hz). The filtered signals were sampled and transmitted via Bluetooth at 54 Hz to a smartphone running a custom Android application, where they were stored for further processing.

The IMU chips are factory-calibrated. A central requirement of the wearable system is its “plug and play” character; therefore, no further in-field sensor calibration was performed. While we understand that changes in environmental conditions could influence the measurements, we could not discern any differences between experiments and between individual systems, suggesting that external conditions have negligible impact. In addition, GNSS signals of a mobile phone (iPhone 6) were recorded at a sampling rate of 1 Hz.

### 2.2. Data

#### 2.2.1. Data Generation

Twenty advanced skiers were recruited to participate in this study. All participants were either ski instructors or competitive alpine skiers, including three former FIS World Cup athletes. Participants were informed of the testing procedures in detail, including possible risks and benefits of the investigation, prior to signing the consent form as approved by the local ethics committee (EK-GZ. 11/2018). This experiment was conducted in accordance with the Declaration of Helsinki.

In order to construct a dataset containing as many skiing styles as possible, participants completed a series of nine skiing runs, performing at least ten consecutive turns of a given style per run. Video footage of each run confirmed that the participants were skiing according to the instructions.

The target turn radii were defined relative to the width of a snowcat track, which is approximately five meters. During the parallel-style runs (carving and drifting), participants completed one run each of long radius turns (≥three times snowcat track width ≈12 m), medium radius turns (≥two times snowcat track width ≈8 m) and short radius turns (<two times snowcat track width). Participants skied a total of six parallel-style runs.

During the non-parallel-style runs, participants performed a pure snowplow run and a snowplow-steering run in which the turn initiation and turning phases were skied in a snowplow, and the completion phase was skied in the parallel technique [8]. Finally, participants completed a “race” run, where participants were instructed to ski at their highest intensity or maximum performance. All trials were supervised by an investigator and repeated if the trial was not completed according to instructions.

All data collection occurred between January and March 2019 at three Austrian ski resorts. In order to control for slope conditions, all data collection took place on blue or red slopes. Data were collected in various snow conditions including freshly groomed, icy, soft and up to 10 cm of fresh snow. Table A5 in the Appendix A gives an overview of the snow conditions during data collection. Although not conducted in a systematic manner, this provides a diverse dataset of skiing with which to train a robust classifier.

All participants skied on the same commercially available recreational race skis. Long and medium radius turns were performed on giant slalom skis (Atomic Redster G9, 171/177/183 cm length, 18.6 m radius). Short radius and non-parallel turns were performed on slalom skis (Atomic Redster S9, 155/165 cm length, 12.7 m radius). Participants completed 2–3 runs on each ski prior to testing to familiarize themselves with the test skis.

#### 2.2.2. Data Pre-Processing

The IMU hardware assigned a relative timestamp (i.e., milliseconds since power on) to each data point. In order to synchronize left and right IMU, the smartphone replaced the original timestamp with the phone’s timestamp at the time of data arrival minus some offset. This offset was half of the Bluetooth connection’s round-trip time, which was continuously re-calculated to avoid clock drift. With both IMU and GNSS data on the same timeline, the application resampled the IMU data together with the speed values at 54 Hz with linear interpolation. In case one of the two IMUs lost connection, the data of the still operational IMU were treated as both the left and right signal until the connection was automatically re-established. If both IMU lost connection simultaneously, the application stopped recording after 60 missed values (~1.1 s). Finally, one trial produced one labeled, multivariate time series of IMU data from both ski boots, synchronized to an absolute timestamp. For further analysis, the data from both accelerometer and gyroscope were filtered with a zero-lag, 4th order, low-pass Butterworth filter with a cutoff frequency of 6 Hz. This filter cutoff was chosen in order to maintain 95% of the signal power.

**Figure 2:**
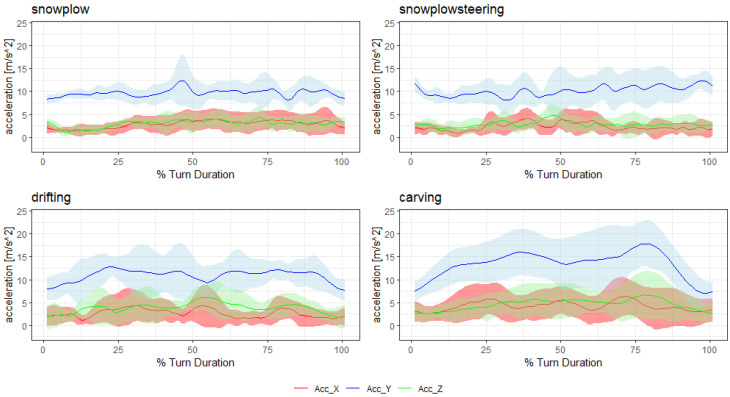
Accelerometer data for each style.

**Figure 3:**
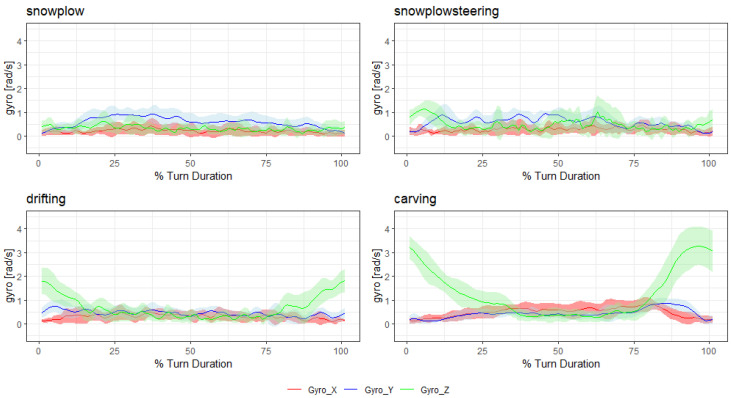
Gyroscope data for each style.

To give an idea of the signal shape for each style, Figure 2 and Figure 3 show the mean and standard deviation of the time normalized filtered absolute accelerometer and gyroscope signal of all turns within a run (without the first two “warm-up” turns). The data for these figures were extracted from one exemplary subject.

**Figure 4:**
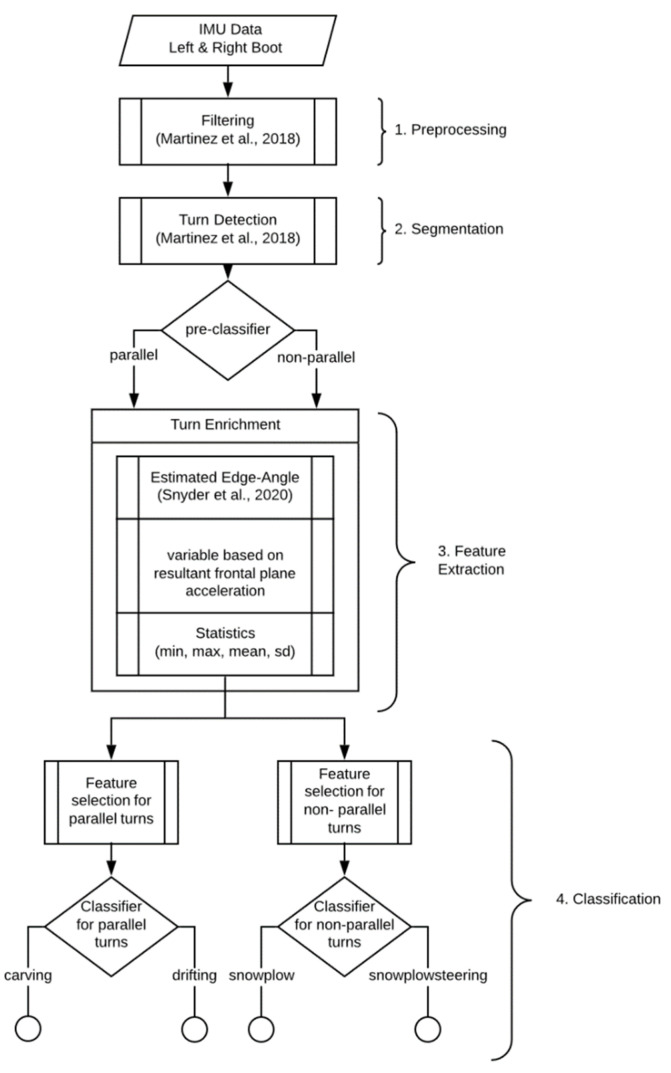
Flowchart from raw data to classification.

With respect to the extended activity recognition chain [11], these time series were pre-processed and segmented into individual turns before classification. This was implemented according to Martínez et al. [3,18]. This turn detection algorithm was designed specifically for alpine skiing and is not only accurate and precise but can process live data streams, meaning that turns can be automatically detected during the actual skiing activity without any additional action from the user. The first and last turn of each run were discarded to eliminate potentially atypical acceleration or deceleration. The final enriched and pre-processed data set used for classification consisted of 2063 individual turns (851 drifting, 920 carving, 201 snowplow and 91 snowplow-steering), each with 57 features (after feature extraction; see Section 2.3.2). Figure 4 illustrates the entire classification process.

#### 2.2.3. Training and Testing Data

**Figure 5:**
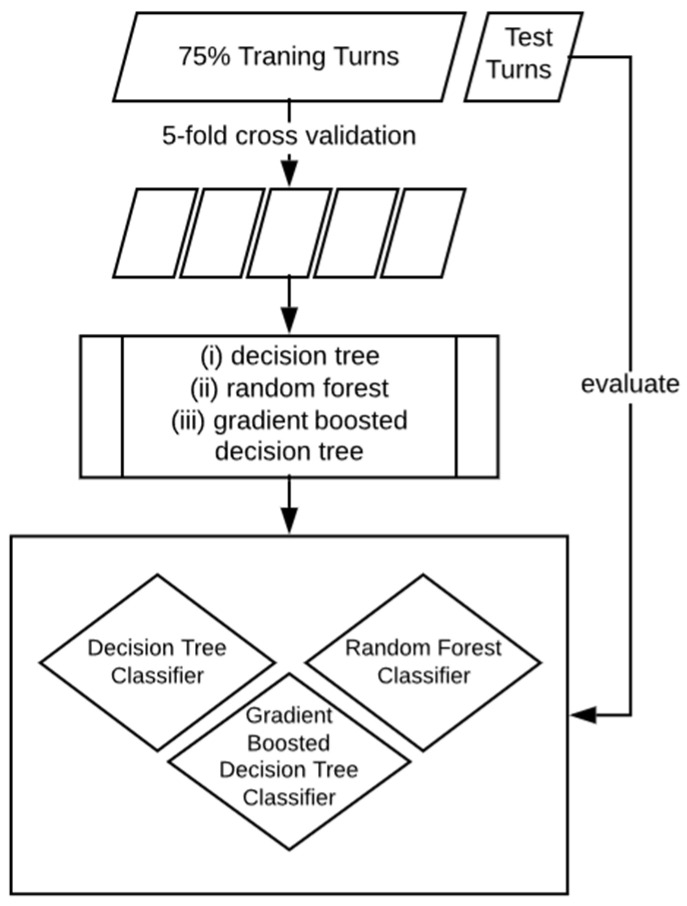
Training and testing data set.

In order to train the classifiers and evaluate their performance, the enriched turn data were split into two subsets (see Figure 5). A total of 75% of all turns were used as the training set with which the models were generated using 5-fold cross validation. The remaining 25% served as test data to validate the trained model, discover possible overfitting and compare the final classification models. The turns were assigned randomly to either the training or the testing data set. As participants were a relatively heterogeneous mix of experts, runs were split record-wise in order to include a high number of heterogeneous participants and in order to avoid an underfitting of the model and a high classification error (see [26,27,28] for discussions on the topic of data splits).

### 2.3. Methods

#### 2.3.1. Pre-Classification into Parallel and Non-Parallel

**Figure 6:**
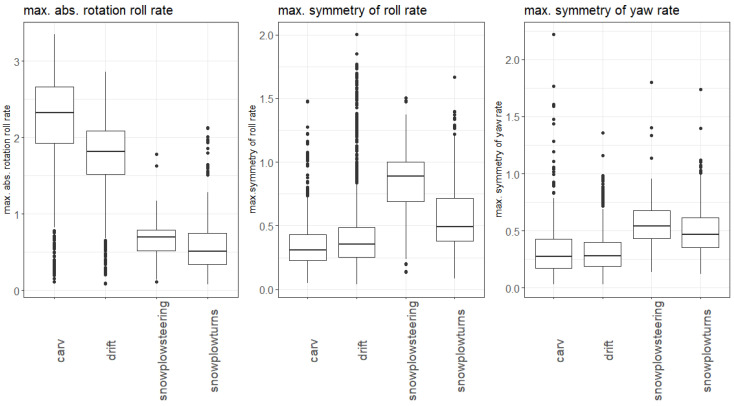
Features for the parallel/non-parallel distinction. The features are visualized via boxplots which show the summary statistics. The box represents the interquartile range (IQR = third quartile–first quartile), the thick line the median. The whiskers show the minimum and maximum values without outliers (1.5*IQR), the black dots the outliers which lie outside 1.5*IQR.

For pre-classification of the turn into parallel and non-parallel, a single decision tree was built with three features selected based on domain knowledge and visual data analysis over all available features: the maximum symmetry of the roll axis angular velocity, the maximum symmetry of the yaw axis angular velocity and the maximum absolute roll axis angular velocity. Figure 6 shows the distribution of these three features dependent on the skiing style. The boxplots indicate that the maximum roll axis angular velocity was much higher for parallel (carved and drifted) than for non-parallel turns (snowplow-steered and snowplow). The maximum symmetry of the yaw and roll axis angular velocity was on average smaller for parallel turns than for non-parallel.

**Figure 7:**
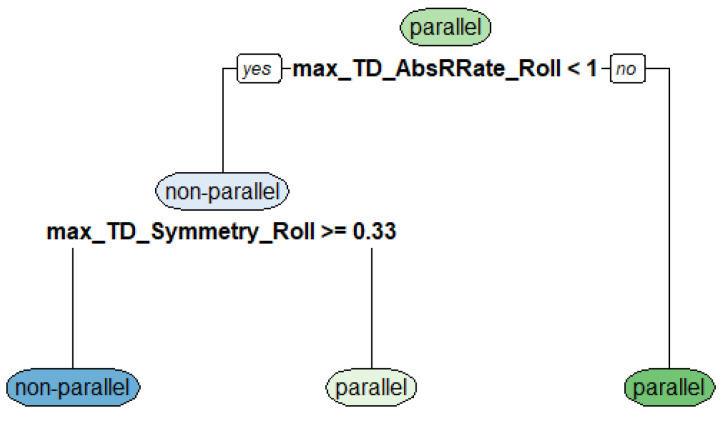
Decision tree for pre-classification (max_TD_AbsRate_Roll = maximum absolute roll axis angular velocity, max_TD_Symmetry_Roll = maximum symmetry of the roll axis angular velocity).

After training the decision tree with 75% of the training data, the best model was determined to be a two-knot tree based on two final features: maximum absolute roll axis angular velocity (max_TD_AbsRate_Roll) and the maximum symmetry of the roll axis angular velocity (max_TD_Symmetry_Roll). Testing the tree with the 25% remaining test data resulted in an accuracy of 95.85%. Figure 7 shows the decision tree of the pre-classification model visually.

#### 2.3.2. Feature Extraction

As visualized in Figure 4 several features were extracted from each turn’s filtered and unfiltered accelerometer and gyroscope signals. The extracted features can be broadly categorized into (i) statistics (mean, max, min, standard deviation) of the average filtered and unfiltered signal of the left and right IMU combined (e.g., the maximum gyroscope roll axis angular velocity of the turn), (ii) symmetry (i.e., absolute distance) between the left IMU and right IMU signal, (iii) features estimated from the phone’s GPS (speed and turn size) and (iv) skiing specific features, such as inclination angle [12] and resultant acceleration. Further descriptions of the features extracted and used for classification are listed in Table A2 and Table A3 in the Appendix A. The features extracted for this model are limited to those which can be calculated online during the feature extraction step (step 3, Figure 4) of the activity recognition chain.

#### 2.3.3. Feature Selection

In order to decrease the number of features that were used to develop the following skiing style classification models, a preliminary feature selection was applied. Starting with a candidate feature set of 57 features per turn, we performed recursive feature elimination in combination with random forests, as explained by Granitto et al. [29]. In recursive feature elimination, the model is first fit with all candidate features. Then, the features are sorted by their importance in the model. At each iteration of the algorithm, only the top ranked features are kept and the model is refit with this reduced set of candidate features. In this work, models of the size 1, 2, 3, 4, 5, 10, 15 and 20 and all features as input variables were tested and compared via the accuracy metric on a cross-validated data set.

#### 2.3.4. Classification Methods

This work compares three different classification approaches and focuses on tree-based algorithms for the classification of the alpine skiing style. In order to identify important features, we focused on algorithms whose explanation and interpretation degree are higher than in deep learning models [30,31]. The proposed methods are either directly explainable or interpretable post-hoc [31], which can therefore identify the distinction of alpine skiing styles’ important features: (i) decision trees [32], (ii) random forest [33] and (iii) gradient boosted decision trees [34].

Gupta et al. [35] mention advantages of decision trees, such as the easy interpretability and visualization, the possibility to handle categorical as well as numerical outcomes and the little data preparation that is required.

The random forest developed by Leo Breiman [33] is a bagging method and consists of multiple independent trees. Each tree is grown randomly by a bootstrapped sample of the training set. For each node, a subset of features is selected at random. Their best split is used to split the node [33].

The main idea of boosting is to combine a lot of weak classifiers and increase the accuracy due to this combination [36]. Boosted decision trees are a combination of a lot of weak decision trees and are explained by Hastie et al. [34]. Random forests and gradient boosted trees are slower to construct, but they are usually more robust and have better performance than single decision trees [37].

We generated three models using each of these learning algorithms and our training data set. To generate the simple decision tree, we used a recursive partitioning algorithm [38] which is mainly based on the classification and regression tree (CART) algorithm [32]. For the gradient boosted decision trees, the extreme gradient boosting (xgboost) algorithm [39] was applied.

Several parameters of the learning algorithms, such as the number of trees in a random forest, have to be chosen before model training. In order to find the best parameter setup for each model, cross-validation was used and the model parameters with the highest mean accuracy of the folds were chosen. Table A4 in the Appendix A lists the parameters used for all three algorithms. The feature selection as well as the model fitting were applied separately for parallel and non-parallel turns.

#### 2.3.5. Performance Measures

In order to compare the classification performance of the models, four metrics for comparison were used: Accuracy, sensitivity, specitivity and the geometric mean. Furthermore, confusion matrices for a visual interpretation of the results are provided. In the alpine skiing ski style classification, two outcome classes for each of the two style classification problems exist. An unknown turn can be thus, if parallel, classified into drifting or carving or, if non-parallel, into snowplow-steering or snowplow. As the turn may be classified either correctly or incorrectly, four possible outputs exist for each of the two classification problems. Table 1 and Table 2 show the confusion matrices for the two classification cases in general.

Table 3 summarizes the performance metrics that were used for model comparison of the classifications numerically. Performance measures for classfication problems are described in detail by [40,41,42]. In the field of sports analytics, the most commonly used performance metric is accuracy [43]. In addition to accuracy, we also calculate sensititvity and specificity and report the geometric mean, as this measure is less sensitive to imbalanced data than other metrics [42].

### 2.4. Software

The algorithms and methods of this paper were calculated with the statistical software R [44]. The algorithms and libraries used for this analysis are listed in Table A1 in the Appendix A of this work.

## 3. Results

### 3.1. Feature Selection

**Figure A1:**
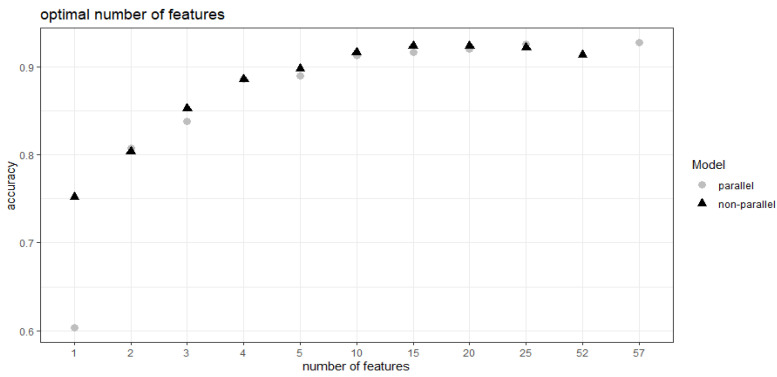
Optimal number of features for the parallel and non-parallel model.

The recursive feature elimination of the parallel turns showed that the accuracy of the cross-validated data set increased depending on the number of input features from 0.603 for one feature to 0.928 for all 57 features. As there was no large increase in accuracy between 25 and 57 features used (0.001 points—See Figure A1 in the Appendix A), the final prediction model for the parallel turns was calculated with the most important 25 features, which are listed in Table A2 in the Appendix A of this article.

In the case of the non-parallel turns, the recursive feature elimination process showed (see Figure A1 in the Appendix A) that accuracy was highest with 0.923 in the model containing 20 explanatory features and lowest with 0.752 for the single feature model. The list of the 20 features used for the classification of non-parallel turns is attached in Table A3 in the Appendix A.

### 3.2. Important Features for Classifcation of the Alpine Skiing Styles

**Figure A2:**
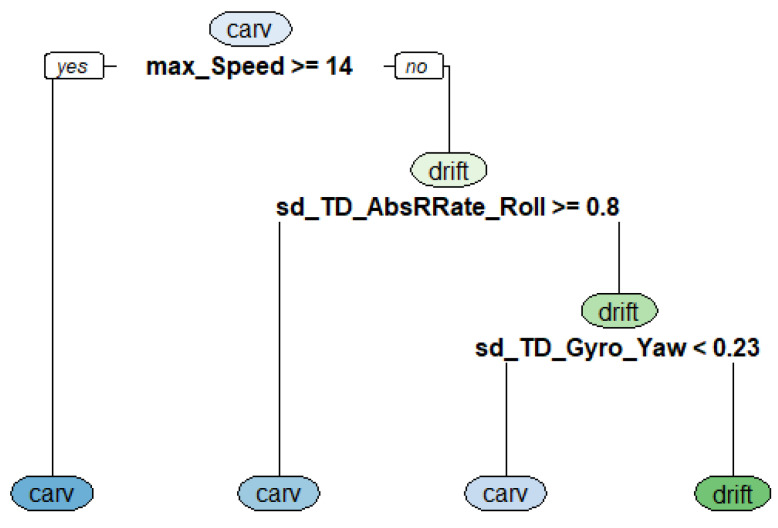
Decision tree for the classification of the parallel turns (max_Speed = maximum speed of turn, sd_TD_AbsRRate_Roll = standard deviation of absolute roll axis angular velocity of turn, sd_TD_Gyro_Yaw = standard deviation of gyroscope roll axis angular velocity of the turn).

**Figure A3:**
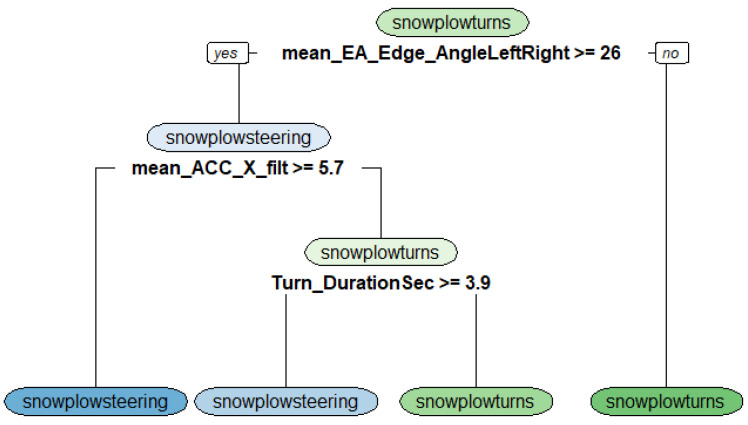
Decision tree for the classification of the non-parallel turns (mean_EA_Edge_AngleLeftRight = mean of the maximum estimated inclination angle of the left and right foot of turn, mean_ACC_X_filt = mean of the maximum of the acceleration of the *X*-axis of left and right foot of turn, Turn_DurationSec = duration of turn (in seconds)).

The most important variables of the final decision tree for the parallel classification task were the maximum speed per turn, the standard deviation of the absolute roll axis angular velocity of the turn and the standard deviation of the gyroscope roll axis angular velocity of the turn. The non-parallel classification tree consisted of the mean of the maximum estimated inclination angle of the left and right foot of the turn, the mean of the maximum of the acceleration of the *X*-axis of left and right foot of the turn and the turn duration (see Figure A2 and Figure A3 in the Appendix A).

**Figure 8:**
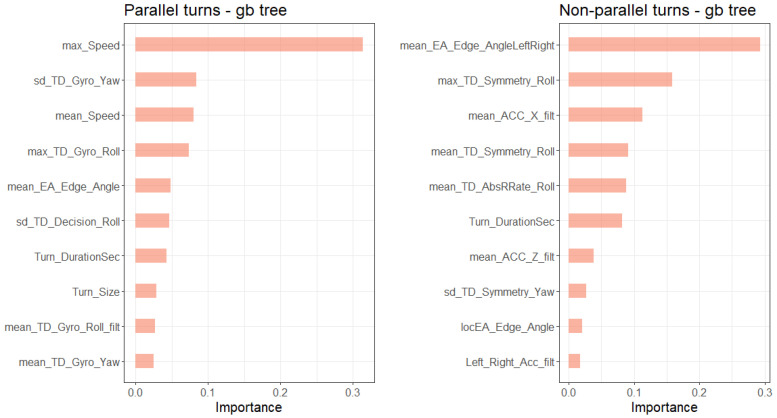
Variable importance plot of the 10 most important variables of the gradient boosted tree.

**Figure 9:**
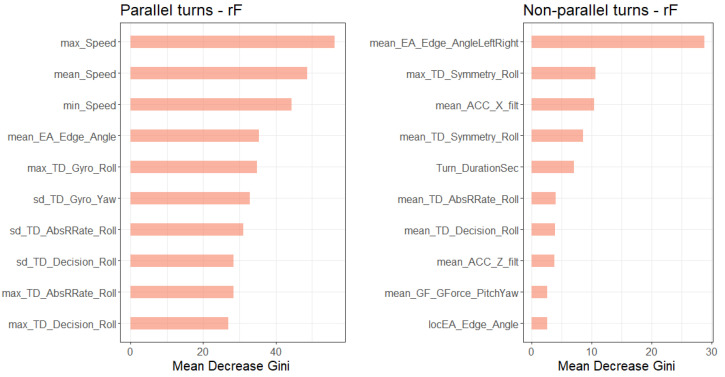
Variable importance plot of the 10 most important variables of the random forest.

Figure 8 and Figure 9 show the 10 most important features for ski style classification of the gradient boosted tree and the random forest based on importance metrics. These metrics display the contribution of each variable based on the total gain of this variable’s splits and the mean decrease in Gini impurity (see [39,45] for descriptions of the metrics). The most important features for the classification models of the parallel turns were speed (maximum, minimum and mean of each turn), the standard deviation of the yaw axis angular velocity and the maximum gyroscope roll axis angular velocity of the turn.

For the non-parallel turn classification, the most important features in the models were the mean estimated inclination angle, the maximum symmetry of the roll axis angular velocity of the turn and the mean yaw axis angular velocity of the acceleration.

### 3.3. Comparison of Model Performance

Table 4 and Table 5 summarize the performance metrics of the three models. In the parallel case, the random forest and the gradient boosted decision tree performed similarly well. In the non-parallel case, the accuracy for the test set ranged from 0.822 to 0.890 and the geometric mean from 0.769 to 0.807. Again, as in the non-parallel case, the performance of the random forest and the boosted decision tree was similar, but sensitivity was smaller than specificity. This implies that the two non-parallel skiing styles were not predicted equally well.

Table 6, Table 7, Table 8, Table 9, Table 10 and Table 11 summarize the results of the decision tree, the random forest and the gradient boosted decision tree prediction with confusion matrices visually.

The classification of the parallel turns into carving and drifting with random forest and gradient boosted decision tree showed similar results. The random forest predicted 227 out of 242 carved and 193 out of 201 drifted turns correctly. The gradient boosted tree explained 232 out of 242 carved and 190 out of 201 drifted alpine skiing turns.

In the non-parallel classification task, the performance of the random forest and the gradient boosted tree were again similar. The random forest predicted 11 out of 16 snowplow-steering and 54 out of 57 snowplow turns correctly. The gradient boosted tree also explained 11 out of 16 snowplow-steering and 53 out of 57 snowplow turns.

## 4. Discussion

### 4.1. Classification Performance

The simplest method, a decision tree, is easily interpretable but performed worse compared to the other two classification methods applied in the current study. Both random forest and gradient boosted classification trees achieved accuracies of more than 93% in the parallel classification task (drifted versus carved) and 88% in the non-parallel case (snowplow versus snowplow-steering). This classification accuracy might be improved by considering slope and weather conditions. One further reason for the lower classification accuracy of the non-parallel turns may be due to the smaller sample size of the non-parallel turns available for the training of the algorithm.

Within the class of parallel turns, the drifted and carved turns were equally well predictable. As for non-parallel turns, the snowplow and snowplow-steered turn classifier showed smaller sensitivity than specificity, indicating that snowplow-steered turns were predicted more accurately than snowplow turns. Looking at individual misclassified turns did not imply a unique reason for turn style misclassification. Some misclassified carving turns that were incorrectly classified as drifted turns were slower and smaller turns than the corrected classified carved turns. On the other hand, some misclassified drifted turns that were misclassified as carved showed higher mean estimated inclination angle values than the corrected classified drifted turns. Additionally, in the non-parallel case, misclassified snowplow-steered turns that were wrongly classified as snowplow turns had lower mean and maximum estimated inclination angle values compared to correctly classified snowplow-steered turns.

### 4.2. Limitations

Although the classification models achieved an accuracy between 88.5% and 95.3% for parallel and 82.2% and 89.0% for non-parallel turns, the compared methods have disadvantages. Decision trees based on the CART algorithms may be unstable and split only by one variable [35]. Random forests, on the other hand, are more robust than decision trees but may be slow to construct [37]. Additionally, gradient boosted trees are slow to train and may suffer from overfitting [37].

Currently, the classification is based on a two-step process where the turn is first classified into parallel or non-parallel and then classified again into the detailed skiing styles. Further research may test and compare a one-step classification where the final skiing style is classified in a single step for a more general model.

As reported in the Methods section, the data for the trained model were generated by a limited number of participants and under controlled skiing conditions. Therefore, this algorithm is adequately prepared to classify skiing performed on moderate slopes with limited fresh snowfall. In order to validate the model with other conditions, such as powder snow, bumpy or mogul slopes, the model would need to be tested on additional data sets containing well labeled data from those conditions. In the same context, it is not yet known whether this model will be able to accurately classify skiing styles on various other slopes, such as flatter beginner slopes or extremely steep expert slopes.

As the turns included in this study were generated by intermediate and expert skiers, the classifier has only been validated for those skier abilities. Therefore, we suggest further validation of the algorithm on datasets containing additional skier abilities, including beginner skiers, that also can test the current trained model of possible overfitting.

In order to check the robustness of the suggested models, different random seeds for the splitting of the turns into training and testing data were set, and new models, based on these different random seeds, were developed with the different training sets. These models showed very similar classification performances for the parallel classification. Accuracies were lowest for the decision trees and highest for the gradient boosted trees. The random forest and gradient boosted tree showed accuracies between 92% and 94% for the parallel classification. Additionally, for the non-parallel classification, task accuracies were lowest for the decision tree. However, the accuracies of the non-parallel turns varied between 87% and 95% depending on the different seeds, which is why we conclude that the model for the non-parallel classification is not as robust as the parallel classification model. Although accuracies of the non-parallel classification were always larger than 87%, the models performed worse and were more unstable than the models for the parallel classification. We therefore suggest additional data collection of especially non-parallel skiing turns (snowplow-steering and snowplow) as additional observations of non-parallel turns may improve the model accuracies for the non-parallel classification.

Furthermore, instead of considering an isolated, single and independent turn for classification, it might be beneficial for the prediction accuracy to also consider recent turns when classifying the current turn and to account for dependencies between the turns. Applying deep learning methods, such as a convolutional neural network or a long short-term memory network [46] may also improve classification performance.

### 4.3. Application of the Classifier

The results of the proposed classifier are suitable for implementation within a framework such as the extended activity recognition chain (eARC) proposed by Brunauer and colleagues [11]. Previous work by this group has already implemented the preprocessing, segmentation [3,18] and feature extraction [12] steps of the eARC. This work presents a concrete example of the eARC’s fourth step-classification-and by doing so furthers the development of a data-driven, automated motor training and coaching system, as suggested by Kos et al. [47]. All algorithms used in the proposed processing chain are capable of analyzing live data streams and produce information about individual turns with little delay.

### 4.4. Sensor Setup

The sensor setup is simple, uses widely available hardware and can be replicated with low cost. It requires neither calibration routines nor any other special attention from the user. However, the proposed classification method uses two IMU sensors simultaneously for capturing left and right boot dynamics, respectively. This setup was able to capture symmetry information, which was shown to be an important factor for classifying the non-parallel styles. Future work could further investigate the relationship of the left and right ski and its impact on skiing performance.

## 5. Conclusions

We have presented a classifier based on GNSS and IMU data that is able to distinguish turns into four ski turn styles: drifting and carving (i.e., parallel turns) and snowplow and snowplow-steering (i.e., non-parallel turns). Overall, the gradient boosted decision trees produced slightly better models than the random forest. The prediction accuracy of the best models is over 95% and 89% for the classification of parallel and non-parallel turns, respectively. Nevertheless, we recommend further research that validates the accuracy of the boosted classification model with respect to different snow and slope conditions and various skill levels of the skiers.

## Figures and Tables

**Table 1:** Confusion matrix for parallel turns.

Parallel Turns		Actual	
		Drifting	Carving
**Predicted**	Drifting	True drifting turns (tp)	False carving turns (fn)
	Carving	False drifting turns (fp)	True carving turns (tn)

**Table 2:** Confusion matrix for non-parallel turns.

Non-Parallel Turns		Actual	
		Snowplow	Snowplow-Steering
**Predicted**	Snowplow	True snowplow turns (tp)	False snowplow-steering turns (fn)
	Snowplow-Steering	False snowplow turns (fp)	True snowplow-steering turns (tn)

**Table 3:** Performance metrics for model comparison (for the abbreviations, please see Table 1 or Table 2).

Metrics	Formula
Accuracy (acc)	$Accuracy=\frac{tp+tn}{tp+tn+fp+fn}$
Sensitivity (sn)	$Sensitivity=\frac{tp}{tp+fn}$
Specificity (sp)	$Specificity=\frac{tn}{tn+fp}$
Geometric mean	$Geometric\;Mean=\sqrt{sn*sp}$

**Table 4:** Performance measures for classification of parallel turns on test data set.

	Accuracy	Sensitivity	Specificity	Geometric Mean
Decision Tree	0.885	0.901	0.866	0.883
Random Forest	0.948	0.938	0.960	0.949
Boosted Tree	0.953	0.959	0.945	0.951

**Table 5:** Performance measures for classification of non-parallel turns on test data set.

	Accuracy	Sensitivity	Specificity	Geometric Mean
Decision Tree	0.822	0.688	0.860	0.769
Random Forest	0.890	0.688	0.947	0.807
Boosted Tree	0.877	0.688	0.930	0.800

**Table 6:** Confusion matrix for parallel turns—Decision tree.

Parallel Turns		Actual	
		Carving	Drifting
**Predicted**	Carving	218 (90.1%)	27 (13.4%)
	Drifting	24 (9.9%)	174 (86.6%)

**Table 7:** Confusion matrix for parallel turns—Random forest.

Parallel Turns		Actual	
		Carving	Drifting
**Predicted**	Carving	227 (93.8%)	8 (4.0%)
	Drifting	15 (6.2%)	193 (96.0%)

**Table 8:** Confusion matrix for parallel turns—Gradient boosted tree.

Parallel Turns		Actual	
		Carving	Drifting
**Predicted**	Carving	232 (95.6%)	11 (5.5%)
	Drifting	10 (4.1%)	190 (94.5%)

**Table 9:** Confusion matrix for non-parallel turns—Decision tree.

Non-Parallel Turns		Actual	
		Snowplow-Steering	Snowplow
**Predicted**	Snowplow-Steering	11 (68.8%)	8 (14.0%)
	Snowplow	5 (31.2%)	49 (86.0%)

**Table 10:** Confusion matrix for non-parallel turns—Random forest.

Non-Parallel Turns		Actual	
		Snowplow-Steering	Snowplow
**Predicted**	Snowplow-Steering	11 (68.5%)	3 (5.3%)
	Snowplow	5 (31.2%)	54 (94.7%)

**Table 11:** Confusion matrix for non-parallel turns—Gradient boosted tree.

Non-Parallel Turns		Actual	
		Snowplow-Steering	Snowplow
**Predicted**	Snowplow-Steering	11 (68.5%)	4 (7.0%)
	Snowplow	5 (31.2%)	53 (93.0%)

## Appendix A

Table A1 lists the R packages and their versions (without package dependencies) used in this analysis. R was applied in version 3.6.1. [44].

**Table A1:** R packages.

R Package Name	Version
caret [48]	6.0–84
data.table [49]	1.12.2
tidyr [50]	1.0.0
dplyr [51]	0.8.3
packrat [52]	0.5.0
randomForest [45]	4.6–14
xgboost [39]	0.90.0.2
rpart [38]	4.1–15
rpart.plot [53]	3.0.8
ggplot2 [54]	3.2.1

Table A2 and Table A3 list the features used for alpine skiing style classification. Table A2 contains the used features for the prediction of drifted and carved turns based on recursive feature elimination. Table A3 lists the features for the prediction of the snowplow-steered and snowplow turns.

**Table A2:** List of features for drifted and carved turn prediction.

Feature	Description
sd_TD_Gyro_Yaw	Standard deviation of gyroscope roll axis angular velocity of turn (mean of left and right boot) (rad/s)
max_Speed	Maximum speed of turn (m/s)
mean_Speed	Mean speed of turn (m/s)
max_TD_Gyro_Roll	Max gyroscope roll axis angular velocity of turn (mean of left and right boot) (rad/s)
min_Speed	Minimum speed of turn (m/s)
mean_EA_Edge_Angle	Mean estimated inclination angle of turn (mean of left and right boot) (rad/s)
Turn_DurationSec	Duration of turn (s)
sd_TD_Gyro_Roll	Standard deviation of gyroscope roll axis angular velocity of turn (mean of left and right boot) (rad/s)
sd_TD_Decision_Yaw	Standard deviation of filtered (using a fourth-order, zero-lag, low-pass Butterworth filter with cut-off frequency of 0.5 Hz) yaw axis angular velocity of turn (mean of left and right boot) (rad/s)
max_TD_Gyro_Yaw	Maximum yaw axis angular velocity of turn (mean of left and right boot) (rad/s)
mean_GYRO_Z_filt	Mean of the maximum of the gyroscope of the *Z*-axis of left and right foot of turn (rad/s)
max_TD_AbsRRate_Roll	Maximum absolute roll axis angular velocity of turn (mean of left and right boot) (rad/s)
max_TD_Decision_Roll	Maximum filtered (using a fourth-order, zero-lag, low-pass Butterworth filter with cut-off frequency of 0.5 Hz) roll axis angular velocity of turn (mean of left and right boot) (rad/s)
Turn_Size	Size of turn
sd_TD_AbsRRate_Roll	Standard deviation of absolute roll axis angular velocity of turn (mean of left and right boot) (rad/s)
sd_TD_Decision_Roll	Standard deviation of filtered (using a fourth-order, zero-lag, low-pass Butterworth filter with cut-off frequency of 0.5 Hz) roll axis angular velocity of turn (mean of left and right boot) (rad/s)
mean_TD_Gyro_Roll_filt	Mean gyroscope roll axis angular velocity of turn (mean of left and right boot) (rad/s)
max_TD_Decision_Yaw	Maximum filtered (using a fourth-order, zero-lag, low-pass Butterworth filter with cut-off frequency of 0.5 Hz) yaw axis angular velocity of turn (mean of left and right boot) (rad/s)
max_TD_Symmetry_Roll	Maximum symmetry of the roll axis angular velocity of turn between left and right boot (rad/s)
mean_GYRO_Y_filt	Mean of the maximum of the gyroscope *Y*-axis of left and right foot of turn (rad/s)
mean_TD_Gyro_Yaw	Mean gyroscope yaw axis angular velocity of turn (mean of left and right boot) (rad/s)
sd_TD_Symmetry_Roll	Standard deviation of the symmetry of the roll axis angular velocity of turn between left and right boot (rad/s)
mean_TD_Gyro_Roll	Mean gyroscope roll axis angular velocity of turn (mean of left and right boot) (rad/s)
max_EA_Edge_Angle	Maximum estimated inclination angle of turn (mean of left and right boot) (degree)
EADiff_Left_Right	Mean difference of the absolute estimated inclination angle of left and estimated inclination angle of right foot of turn (degree)

**Table A3:** List of features for snowplow and snowplow-steered turn prediction.

Feature	Description
Turn_DurationSec	Duration of Turn (s)
mean_TD_Gyro_Roll_filt	Mean gyroscope roll axis angular velocity of turn (mean of left and right boot) (rad/s)
max_TD_Symmetry_Roll	Maximum symmetry of the roll axis angular velocity of turn between left and right boot (rad/s)
max_EA_Edge_Angle	Maximum estimated inclination angle of turn (mean of left and right boot) (degree)
locEA_Edge_Angle	Position (1–100) of the max estimated inclination angle of the turn
mean_EA_Edge_Angle	Mean estimated inclination angle of turn (mean of left and right boot) (degree)
mean_EA_Edge_AngleLeftRight	Mean of the maximum estimated inclination angle of the left and right foot of turn (degree)
mean_ACC_Z_filt	Mean of the maximum of the acceleration of the *Z*-axis of left and right foot of turn (m/s^2^)
mean_ACC_Y_filt	Mean of the maximum of the acceleration of the *Y*-axis of left and right foot of turn (m/s^2^)
mean_ACC_X_filt	Mean of the maximum of the acceleration of the *X*-axis of left and right foot of turn (m/s^2^)
mean_GF_GForce_PitchYaw	Mean resultant frontal plane acceleration of turn (mean of left and right boot) (m/s^2^)
max_GF_GForce_PitchYaw	Maximum resultant frontal plane of turn (mean of left and right boot) (m/s^2^)
mean_GF_GForce_PitchYaw_LeftRight	Mean of the maximum resultant frontal plane acceleration of the left and right foot of turn (m/s^2^)
Left_Right_Acc_filt	Mean of the maximal acceleration of the left and right foot of each turn (m/s^2^)
mean_TD_AbsRRate_Roll	Mean absolute roll axis angular velocity (mean of left and right boot) (rad/s)
mean_TD_Decision_Roll	Filtered (using a fourth-order, zero-lag, low-pass Butterworth filter with cut-off frequency of 0.5 Hz) roll axis angular velocity (mean of left and right boot) (rad/s)
mean_TD_Gyro_Roll	Mean gyroscope roll axis angular velocity of turn (mean of left and right boot) (rad/s)
mean_TD_Symmetry_Roll	Mean symmetry roll axis angular velocity of turn between left and right boot (rad/s)
sd_TD_Symmetry_Roll	Standard deviation of symmetry of the roll axis angular velocity of turn between left and right boot (rad/s)
sd_TD_Symmetry_Yaw	Standard deviation of symmetry of the yaw axis angular velocity of turn between left and right boot (rad/s)

Table A4 lists the parameters used in the classification models.

**Table A4:** List of parameters used in models.

Model	Parameters	
	Parallel Turns	Non-Parallel Turns
Decision tree (package: rpart [38])	cp = 0.05076923.	cp = 0
Random Forest (package: randomForest [45])	mtry= 2, ntree = 1000	mtry= 13, ntree = 1000
Gradient boosted decision tree (package: xgboost [39])	max.depth = 3 eta = 0.4 nrounds = 150 gamma =0 colsample_bytree = 0.68 min_child_weight = 1 subsample = 1	max.depth = 3 eta = 0.3 nrounds = 150 gamma =0 colsample_bytree = 0.6 min_child_weight = 1 subsample = 1

**Table A5:** Gives an overview of the snow conditions during data collection.

ID	Snow Conditions
S01	hard groomed
S03	hard groomed
S04	soft (5 cm new snow)
S05	hardpack
S06	hardpack
S07	soft groomed
S08	soft groomed
S09	soft groomed
S10	hard groomed
S11	hard groomed
S12	hard groomed
S14	hardpack
S15	hardpack
S16	soft (6 cm new snow)
S17	hardpack
S19	hardpack
S20	hardpack
S21	hardpack groomed
S23	ice
S24	refrozen spring snow

Figure A1 displays the accuracy measure for the different subset sizes during the feature selection process for the parallel and non-parallel classification tasks.

Figure A2 and Figure A3 plot the decision tree for the classification of the parallel and non-parallel turns.
